# The pH Sensing Properties of RF Sputtered RuO_2_ Thin-Film Prepared Using Different Ar/O_2_ Flow Ratio

**DOI:** 10.3390/ma8063352

**Published:** 2015-06-09

**Authors:** Ali Sardarinejad, Devendra Kumar Maurya, Kamal Alameh

**Affiliations:** Electron Science Research Institute, Edith Cowan University, Joondalup, WA 6027, Australia; E-Mails: d.maurya@ecu.edu.au (D.K.M.); k.alameh@ecu.edu.au (K.A.)

**Keywords:** thin film, sputtering, metal oxide, pH sensing, electrochemical properties

## Abstract

The influence of the Ar/O_2_ gas ratio during radio frequency (RF) sputtering of the RuO_2_ sensing electrode on the pH sensing performance is investigated. The developed pH sensor consists in an RF sputtered ruthenium oxide thin-film sensing electrode, in conjunction with an electroplated Ag/AgCl reference electrode. The performance and characterization of the developed pH sensors in terms of sensitivity, response time, stability, reversibility, and hysteresis are investigated. Experimental results show that the pH sensor exhibits super-Nernstian slopes in the range of 64.33–73.83 mV/pH for Ar/O_2_ gas ratio between 10/0–7/3. In particular, the best pH sensing performance, in terms of sensitivity, response time, reversibility and hysteresis, is achieved when the Ar/O_2_ gas ratio is 8/2, at which a high sensitivity, a low hysteresis and a short response time are attained simultaneously.

## 1. Introduction

Many chemical and biological processes critically rely on accurate pH measurements. Applications, such as water quality monitoring, blood monitoring, chemical and biological analyses, environmental monitoring and various clinical tests, utilize pH sensors [1,2,3,4]. In particular, monitoring the pH level in biomedical applications can be crucial for successful clinical outcomes. Laboratories, as well as industry, utilize pH as a vital analytical tool to monitor water quality, especially in rivers, lakes and oceans, as well as for the neutralization of treated industrial waste water. 

The most widely used technique for pH measurement is based on the use of conventional glass pH electrodes [5,6]. While glass electrodes display several advantages, such as high sensitivity, long-term stability, high ion selectivity and wide operating range, they have key disadvantages including mechanical fragility, need for wet storage, large size, limited shape and high cost, which make them impractical for some applications, such as Lab-on-chips and pH sensor capsules.

Recently, various metal oxides, such as RuO_2_, IrO_2_, PtO_2_, RhO_2_, TiO_2_, SnO_2_, Ta_2_O_5_ and PdO, have been investigated for use in pH sensing electrodes. Their benefits include insolubility, stability, mechanical strength, electro-catalyst and manufacturing technology [7,8,9,10,11,12,13,14,15,16,17,18,19,20]. Compared with other metal oxides, ruthenium oxide exhibits unique properties, including thermal stability, excellent corrosion resistance, low hysteresis, high sensitivity, and low resistivity. Using screen-printing technology, pH sensors employing thick-film ruthenium oxide pH sensing electrodes have been developed in combination with standard electrolyte-filled Ag/AgCl reference electrodes [21,22,23,24,25]. However, while screen printing is a cost-effective deposition technique for thick film development, it is inaccurate (hence impractical) for the development of sub-micron thin-films. Recently, pH sensor structures based on the use of sub-micron RuO_2_ thin films have been reported [26,27,28,29]. 

In this paper, we thoroughly investigate the effect of the Ar/O_2_ gas ratio on the pH sensing performance of the RuO_2_ thin-film sensing electrode prepared by reactive radio frequency (RF) sputtering in conjunction with an electroplated Ag/AgCl reference electrode. Compared to other deposition techniques, RF sputtering offers unique features, such as (i) it maintains the stoichiometry of the target, by adding O_2_ gas to the Ar process gas; (ii) it enables the thickness of the film to be controlled at nano scale, through controlled deposition rates; and (iii) it produces better surface morphology due to molecule-by-molecule sputtering. These unique features result in a super-Nernstian response. A 300 nm thick RuO_2_ thin film is developed with an Ar/O_2_ gas ratio varying from 10/0 to 7/3, and tested using standard pH buffer solutions of pH 4, pH 7 and pH 10, which typically validate the acidity and alkaline behaviors for commercial pH sensors calibration. Experimental results demonstrate a pH sensor of excellent features, including high sensitivity, fast response time, good stability, reversibility and repeatability. The most stable pH sensing performance is achieved for an Ar/O_2_ gas ratio of 8/2. The optimized pH sensor demonstrates several advantages such as miniaturization, ease of packaging, low cost through the use of micro fabrication processes, ruggedness and disposability.

## 2. Sensor Fabrication and Measurements 

### 2.1. Sensor Fabrication

**Figure 1:**
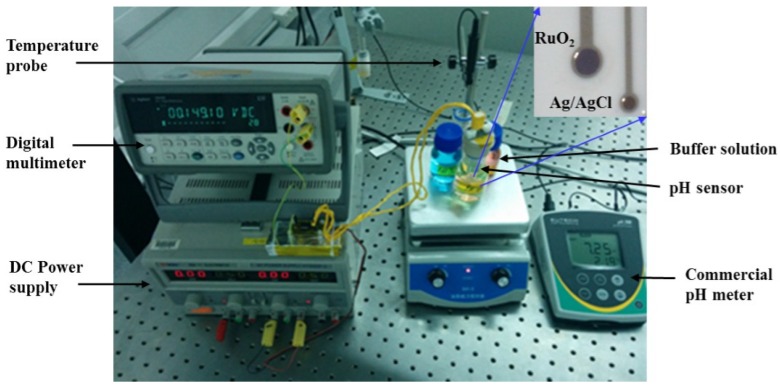
Microphotograph of the pH measurement setup.

The generic structure of the developed pH sensor is shown in Figure 1. The sensor is made-up of an alumina substrate on which two platinum contacts are first deposited. A sub-micron RuO_2_ thin film is radio frequency (RF) sputtered on top of one of the platinum contacts to form the sensing electrode. 

Several pH sensors were developed using off-the-shelf ceramic patterned electrode cells of dimensions 15 mm × 61 mm × 0.67 mm, developed by Pine Research Instrumentation (http://www.pineinst.com), comprising a patterned platinum sensing contact and an electroplated Ag/AgCl reference electrode of sensing diameters 2 mm and 1 mm, respectively, as shown in Figure 1. RuO_2_ thin films were deposited onto the platinum sensing electrode area of the electrode cell utilizing an RF magnetron sputtering system (using shadow masking). A 99.95% pure, 4-inch diameter RuO_2_ sputtering target was used to sputter 300 nm RuO_2_ thin-films in Ar/O_2_ gas ratio varying from 10/0 to 7/3. After pumping down the sputtering chamber to a base presure of 1 × 10^−6^ Torr, Ar and O_2_ gases were introduced into the chamber through mass flow controllers. The deposition process was carried out in pure Ar and Ar/O_2_ gas mixture of ratios ranging from 9/1 to 7/3 at 1 mTorr process pressure and 100 W RF sputtering power. The substrate temperature was kept low, *i.e*., the substrate was not heated during the film deposition. 

### 2.2. pH Measurement 

The potential difference between the electrodes of the pH sensor in real-time was recorded using high performance digital multimeter (Agilent 34410A, http://www.agilent.com). A unity-gain buffer amplifier was developed and used for impedance matching and signal loss minimization. The electrochemical behavior of the sensor was investigated through potentiometric measurements. The potential difference between the RuO_2_ sensing electrode and the Ag/AgCl reference electrode was recorded with 20 s intervals for test solutions of pH 4, pH 7 and pH 10 (Rowe Scientific, Australia) at 22 °C (temperature of our cleanroom). The proof-of-concept experiments were designed to demonstrate the effects of the Ar/O_2_ gas ratio on the performance of the pH sensor. The calibration of commercial pH sensors is typically validated using standard pH values of pH 4, pH 7 and pH 10. Therefore, in this preliminary study, the same buffer solutions were chosen to investigate the performance of the developed pH sensor. A magnetic stirrer was used to continuously stir the test solutions for quicker equilibrium of ion concentrations around the sensing electrodes. As a comparator, test solutions were also measured by an Oakton pH meter (pH 700 benchtop meter), and the sensor was cleaned after each pH measurement using deionized (DI) water rinse followed by N_2_ drying.

## 3. Results and Discussion 

### 3.1. pH Sensing Mechanism

Fog and Buck have proposed five possible interpretations for the pH response mechanism of metal oxides including RuO_2_, with the most accepted interpretation being oxygen intercalation, which is represented by the following equilibrium reaction [14,30]:

MO*_x_* + 2δH^+^ + 2δe^−^ ↔ MO*_x_*_-δ_ + δH_2_O (1)

where δ is the oxygen intercalation, MO*_x_* is a metal oxide with higher oxidation state and MO*_x_*_-δ_ is a metal oxide with lower oxidation state. Note that in Equation (1), oxygen intercalation was assumed with a proton activity in the liquid phase and an oxygen activity in the solid phase. 

Pourbaix diagrams have also been widely adopted for explaining the mechanism governing the redox equilibrium between two insoluble ruthenium oxides, which is represented by the following reaction [31]:

RuO_2_ + 2H_2_O + H^+^ + e^−^ ↔ H_2_O + Ru(OH)_3_ (2)

Up until now, RuO_2_, as an electronically conductive oxide of rutile structure, has been commonly used in electrocatalysis applications [16,17,18]. It is well-known that the high catalytic activity of RuO_2_ is due to several factors, including high surface area, orientation of RuO_2_ molecules, and/or the RuO_2_ nanocrystal structure [19]. Moreover, the effect of surface morphology of ruthenium oxide hydrates has been investigated by measuring the point of zero charge and examining, through cyclic voltammetry, the diffusion of tritium protons in pores, cracks and along grain boundaries [32]. Trasatti [33] has suggested the following general equilibrium for the proton exchange for RuO_2_:

RuO*_x_* (OH)*_y_* + δH^+^ + δe^−^ ↔ RuO*_x_*_-δ_ (OH)*_y_* + δ (3)

The Nernst’s mathematical equation predicting the potential between the sensing and reference electrodes *versus* the pH value is given by [26,27,28]:

(4) $$E=E^{0}-2.303\frac{RT}{F}\text{pH }=\;E^{0}-0.05916\text{ pH}$$

where *E*^0^ is the reference electrode potential, *R* is the gas constant, *T* is absolute temperature and *F* is Faraday’s constant. The entire term $\frac{2.303\;RT}{F}$ is called the Nernst slope. The Nernst slope is 59.16 mV/pH at 25 °C. 

### 3.2. Reactive Sputtering Model 

The growth of the metal-oxide films by sputtering in a reactive environment may be described as follows: When the sputtering target is a metal oxide the oxygen partial pressure is originated from the internal (target) and external sources (gas flow). At steady-state conditions, the flux of oxygen atoms, *f*, is equal to the flux of metal atoms, because of the target homogeneity. In this case, the target supplies molecules into the sputtering chamber with a rate equals to *fA*_t_, where *A*_t_ is the target surface. On the other hand, the external source of oxygen increases the oxygen partial pressure in the chamber by a gain factor or *N*_a_*q*_0_*/V*_0_*,* where *N*_a_ is Avogadro’s number, *V*_0_ *is one mole* volume under normal conditions, *q*_0_ is the oxygen flow. The oxygen concentration inside the chamber decreases because of the oxygen deposition onto both walls of the chamber and the substrate (receiving surface). Using the molecular kinetic theory, the flux of oxygen due to a partial pressure *P* is expressed as:

(5) F=2P/(2πmkT)12

where *m* is the mass of the oxygen molecule, *k* is Boltzmann’s constant and *T* is the absolute temperature.

Note that, as the oxygen gas passes through the system it is removed from the reactive chamber by the system pump at a rate equals to *PSN*_a_*/P*_0_*V*_0_ where *P*_0_ *is* the atmosphere pressure and *S* is the pumping speed. Kissine *et al*. [34] have particularly developed a model for tin oxide (SnO_2_) prepared by varying Ar/O_2_ gas ratio during RF sputtering, showing that, at steady-state condition, the balance of oxygen partial pressure in the vacuum chamber will be governed by the following equation [34]:

(6) q=Ψ(σ+α+ηβ)

where α and β are the parts of the receiving surface covered by Sn and SnO phases respectively, η is a coefficient which shows the relative adsorption of oxygen atoms onto the SnO phase compared to their adsorption onto the Sn phase, *q = 1+q*_0_*N*_a_ */V*_0_*fA*_t_ is the normalized oxygen flow with respect to the flow from the target, σ = *SN*_a_/(2*P*_0_/(2πm*kT*)_1/2_)*V*_0_*A*_s_ is the pumping efficiency, Ψ = *FA*_s_/*fA*_t_, *A*_s_ is the surface of the film deposited on the substrate.

Kissine *et al*. [34] have also made two assumptions, that (i) the surface occupied by the Sn phase (α) increases when Sn is deposited onto the SnO and SnO_2_ phases, however, it decreases because of the oxidation process; and (ii), the surface occupied by the SnO_2_ phase increases when SnO is oxidized, but, it decreases because of the deposition of Sn. Thus, at equilibrium:

(7) $${\text{Ψ}}_{\text{α}}\;=\;\text{β}\;\text{and}\;\text{ηΨβ}\;=\;\text{γ}$$

where γ is the part of the receiving surface covered by the SnO_2_ phase.

Furthermore, Kissine *et al*. [34] have simulated the SnO*_x_* film content *versus* the oxygen flow and concluded that the O:Sn ratio is equal to about 1:1 when the oxygen flow from an external source is rather small, *i.e*., the *q* value is below 1/10, resulting in an amorphous structure. When the oxygen flow (*i.e*., *q*) is increased, the SnO_2_ phase dominates. Note that, however, the crystal perfection and the presence of oxygen vacancies depend on the *q* value. Due to the similarity of Sn and Ru atoms, the above analysis can be used to accurately calculate the stoichiometry of RuO_2_ film prepared under RF sputtering in Ar/O_2_ gas mixture.

### 3.3. pH Sensor Performance

#### 3.3.1. Sensitivity

**Figure 2:**
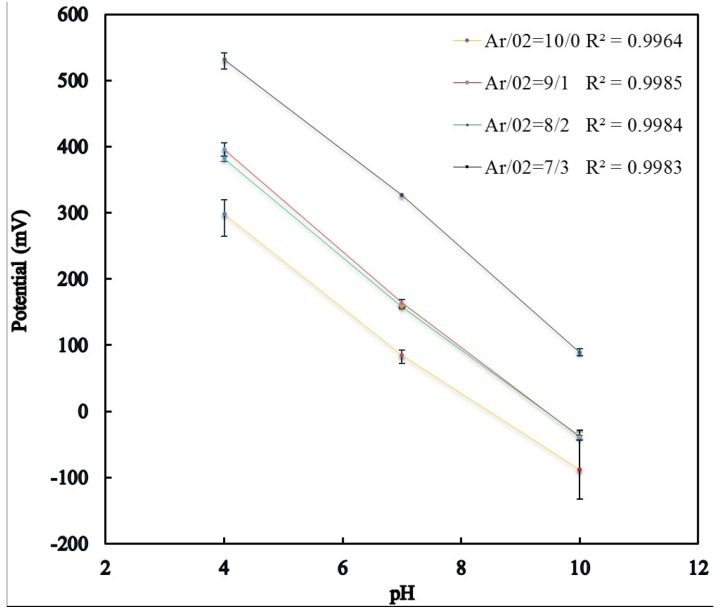
Measured sensor potential *versus* pH for different Ar/O_2_ gas ratios.

The sensitivity of pH sensor was validated by immersing the sensor in pH buffer solution of pH 4, pH 7 and pH 10 at 22 °C. Each sensor was tested three times in the same buffer solution in order to investigate the Nernstian response. Figure 2 shows the average of three tests of the potential *versus* pH values for different oxygen percentages over RuO_2_ sputtering film. The sensor showed super-Nernstian slopes in all cases, starting from 64.33, 72.50, 69.83, and 73.92 mV/pH for Ar/O_2_ gas ratio of 10/0, 9/1, 8/2, 7/3, respectively. The super-Nernstian response of the sensor could be attributed to the use of an RuO_2_ thin-film prepared by RF magnetron sputtering [29,35]. The results shown in Figure 2 demonstrate linear regressions with high correlation coefficient *r*^2^ values between 0.9983 and 0.9964. The pH sensor resolution for different Ar/O_2_ gas ratio was reported as inverse of the sensitivity in the unit of pH/mV.

**Figure 3:**
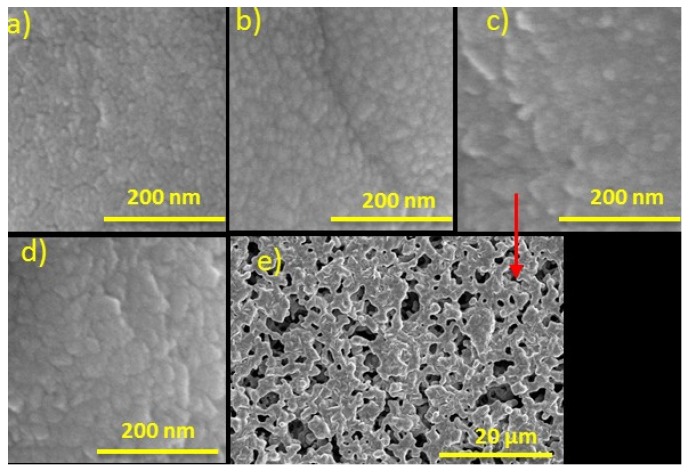
SEM images of the RuO_2_ thin-films for Ar/O_2_ ratios of (**a**) 10/0; (**b**) 9/1; (**c**) 8/2; (**d**) 7/3; (**e**) different magnification for Ar/O_2_ = 8/2.

The increase in sensor potential with decreasing the argon/oxygen ratio could be attributed to a decrease in free carrier density as well as carrier mobility in the RuO_2_ thin film [36]. Cui *et al*. have particularly confirmed that the value for carrier mobility and carrier concentration is very sensitive to the structure of the Indium tin oxide (ITO) films prepared with various Ar/O_2_ gas ratio [37]. Decreasing the Ar/O_2_ ratio or increasing the percentage of oxygen enhances the pore formation in the RuO_2_ thin film thus reducing the carrier density. Furthermore, the enhanced pore formation (or defects) with decreasing the Ar/O_2_ ratio increases the scattering of the charge carrier, thus decreasing the carrier mobility [37]. Furthermore, the RuO_2_ thin-film porosity increases with decreasing the argon/oxygen ratio, thus providing larger sensing surface volume. Figure 3 shows scanning electron microscope (SEM) images of the RuO_2_ thin-films, for different Ar/O_2_ gas ratios. Table 1 shows the pH sensitivity, resolution and linearity of the RuO_2_ sensing film sputtered using different Ar/O_2_ gas ratios.

**Table 1:** pH sensitivity and linearity of RuO_2_ sensing film prepared with different Ar/O_2_ gas ratio.

Ar/O_2_ gas ratio (sccm)	Sensitivity (mV/pH)	Resolution (pH/mV)	Linearity
10/0	64.33	0.015	*r*^2^ = 0.9964
9/1	72.50	0.013	*r*^2^ = 0.9985
8/2	69.83	0.014	*r*^2^ = 0.9984
7/3	73.83	0.013	*r*^2^ = 0.9983

#### 3.3.2. Response Time 

The response time of a potentiometric pH sensor is defined as the transit time required for its potential to reach 90% of an equilibrium value after immersing the sensor in a test solution [5,14]. To measure the response time, the potentials between the electrodes of the developed pH sensors were recorded for 10 min with 20 s intervals for buffer solutions of pH 4, pH 7 and pH 10 at 22 °C for different sensors employing RuO_2_ electrodes developed with various Ar/O_2_ gas ratios in the range 10/0 to 7/3. The response time for each sensor was calculated from the average of three test runs. For an Ar/O_2_ gas ratio of 10/0, the measured response times for pH 4, pH 7 and pH 10 were 3 s, 3 s and 160 s, respectively. For an Ar/O_2_ gas ratio of 9/1, the measured response times for pH 4, pH 7 and pH 10 were 3 s, 3 s and 60 s, respectively. However, when the Ar/O_2_ gas ratio varied from 8/2 to 7/3, the developed sensor displayed a much faster response time of 3 s for all the tested pH values of pH 4, pH 7 and pH 10. Note that, for the Ar/O_2_ ratios 8/2 and 7/3, the measured potential value reached 90% of the equilibrium potential value before the first 3 s of the 20 s interval. For the Ar/O_2_ ratios 10/0 and 9/1, the response time was not 3 s for all tested pH values. Table 2 shows the response time *versus* the Ar/O_2_ ratios for different pH values. Moreover, no change in the response time was displayed when the Ar/O_2_ gas ratio of the RuO_2_ film was further reduced below 7/3. These experiments demonstrated the importance of optimizing the Ar/O_2_ gas ratio for minimizing the response time of the pH sensor. Table 2 shows the response time *versus* the Ar/O_2_ ratios for different pH values.

**Table 2:** Response time *versus* the Ar/O_2_ ratios for different pH values.

Ar/O_2_ gas ratio (sccm)	Response time (s)		
	pH 4	pH 7	pH 10
10/0	3	3	160
9/1	3	3	60
8/2	3	3	3
7/3	3	3	3

Furthermore, the pH sensor exhibited a fast response time for acidic solutions, however, for an alkaline solution (pH 10) the response time of the pH sensor was dependent on the porous properties of the RuO_2_ sensing film, hence, on the Ar/O_2_ gas ratio. For an Ar/O_2_ gas ratio of 8/2, the average pore size is relatively large, compared to 10/0 Ar/O_2_ gas ratio, making it easier to trap the large-size OH^−^ ions and this decreases the time needed to equilibrate the liquid in the pores of RuO_2_ sensing film. For an Ar/O_2_ gas ratio of 7/3, the response time was similar, however, the hysteresis was higher [38].

#### 3.3.3. Stability 

**Figure 4:**
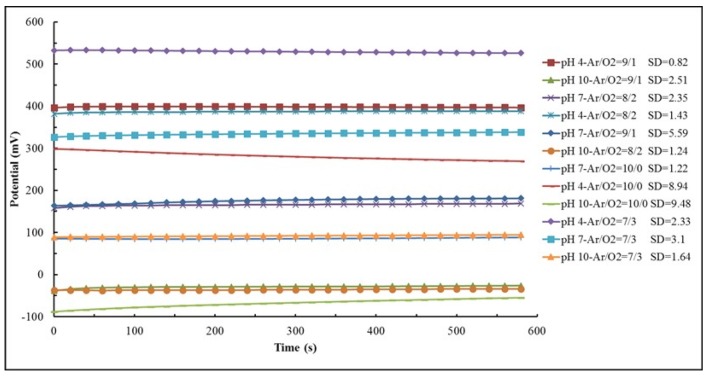
Measured potentials *versus* time in pH 4, pH 7 and pH 10 for different Ar/O_2_ gas ratios (sccm) along with the standard deviation values.

For stability testing, the pH sensor was immersed for 10 min three times in three different test solutions. For each 10 min interval the pH was periodically measured at a sampling time of 20 s. After each 10 min test, the sensor was cleaned with DI water followed by N_2_ drying. Figure 4 shows the measured potential (the average value from the three tests) *versus* time for different pH values. The sensor showed stable output potential for all pH values. The experimental results shown in Figure 4 demonstrate a stable pH sensor’s response for an Ar/O_2_ gas ratio of 8/2. 

#### 3.3.4. Reversibility and Hysteresis

**Figure 5:**
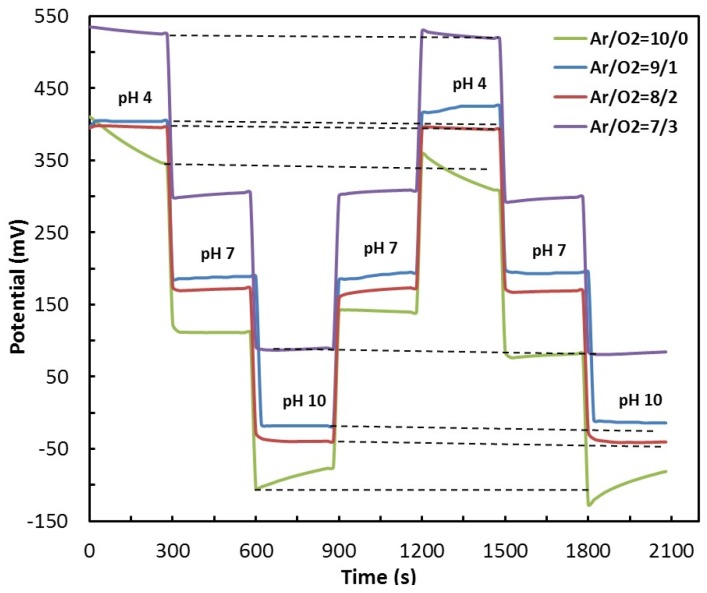
Measured average potential *versus* time when the pH of the solution is sequentially switched between pH 4, pH 7 and pH 10.

To investigate the reversibility of the pH sensor the pH was sequentially switched between pH 4, pH 7 and pH 10 in forward and backward orders at 300 s intervals without cleaning or drying the pH sensor, and the measured potential *versus* time was monitored. The switching cycle was repeated three times. Figure 5 shows the average recorded potentials (averaged over three tests) for different Ar/O_2_ gas ratio in the pH 4, pH 7 and pH 10 loops. The experimental results demonstrate an excellent reversibility and stable pH sensor’s response for an Ar/O_2_ gas ratio of 8/2.

**Figure 6:**
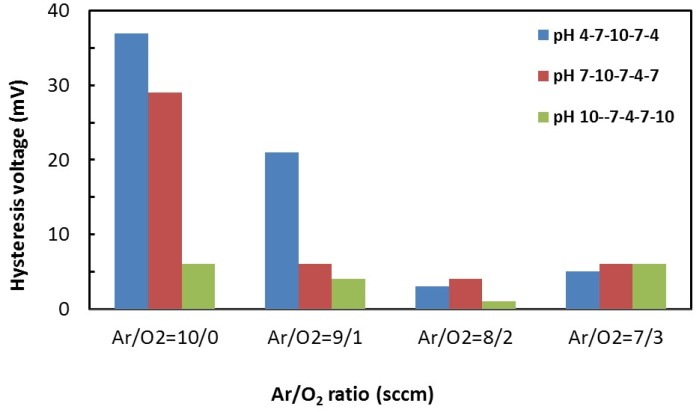
Hysteresis *versus* Ar/O_2_ gas ratios (sccm) for different pH loops.

Typically, when the electrochemical potential of a pH solution is measured several times, different potentials are generated between the electrodes. This phenomenon is called hysteresis and is elaborated elsewhere [27,38]. Figure 6 shows the hysteresis voltage for different Ar/O_2_ gas ratios, for pH 4, pH 7 and pH 10 loops. It is noteworthy to mention that the acid loop hysteresis was smaller for the Ar/O_2_ gas ratio 8/2 compared to that for the Ar/O_2_ gas ratio 10/0. The increase in oxygen percentage increases the porosity of the RuO_2_ film as well as the overall sensing volume, thus resulting in faster ion diffusion through the sensing film. Tsai *et al*. have thoroughly investigated the effect of sensing area on the hysteresis for both acidic and alkaline loops [39].

According to Liao *et al*. [36], the potential different between the pH sensitive film and the electrolyte depends on the pH at the point with zero charge (${\text{pH}}_{\text{pzc}}$). This phenomenon is governed by the site-binding model described by the following equation [39]:

(8) $$2.303\;\left({\text{pH}}_{\text{pzc}}-\text{pH}\right)=\;q\phi_{0\;}/\;KT+\text{sin}h^{-1}\;\left(q\;\phi_{0\;}/KT\text{β}\right)$$

where pHpzc= −log10(Ka×Kb)12= −12( pKa+ pKb) is the pH value at the point of zero charge, ϕ_0_ is the surface potential of the electrolyte/insulator interface with respect to the electrolyte bulk, *K*_a_ and *K*_b_ are equilibrium constants, p*K*_a_= −log (*K*_a_), p*K*_b_= −log (*K*_b_), *K* is the Boltzmann’s constant, *T* is the temperature of the system in Kelvin, β is a dimensionless pH sensitivity parameter, given by [39]:

(9) β=2 q2 Ns (kb/ka)12/KTCDL

where *N*_s_ is the surface site density, and $C_{\text{DL}}$ is the double-layer capacitance and *K*_a_, *K*_b_ are dissociation constants for potential determining ion and counter-ion surface reactions. 

It is evident that the pH sensor structure prepared with an Ar/O_2_ gas ratio of 8/2 results in minimum hysteresis (<4 mV) for all the tested pH range (4–10).

## 4. Conclusions

In this paper, the effect of Ar/O_2_ gas ratio on the performance of RF sputtered RuO_2_ thin-film pH sensors has been experimentally investigated. Several 300 nm thin film RuO_2_ sensing electrodes, prepared by varying Ar/O_2_ gas ratio from 10/0 to 7/3 during RF sputtering, have been developed and their sensitivity, response time, stability, reversibility and hysteresis properties for pH sensing have been investigated. Experimental investigations have shown that an Ar/O_2_ gas ratio of 8/2 results in a RuO_2_ thin-film of excellent pH sensing properties, namely high sensitivity, low hysteresis and faster response, using a conventional RuO_2_ sputtering target. The optimized pH sensor structure has demonstrated a super-Nernstian response of 69.83 mV/pH, good stability and reversibility. The developed pH sensor can further be miniaturized as a lab-on-a-chip device and has application in biological analyses, water quality monitoring, chemical and environmental monitoring and *in vivo* clinical tests.
